# *BnERF114.A1*, a Rapeseed Gene Encoding APETALA2/ETHYLENE RESPONSE FACTOR, Regulates Plant Architecture through Auxin Accumulation in the Apex in *Arabidopsis*

**DOI:** 10.3390/ijms23042210

**Published:** 2022-02-17

**Authors:** Jinyang Lyu, Yuan Guo, Chunlei Du, Haibo Yu, Lijian Guo, Li Liu, Huixian Zhao, Xinfa Wang, Shengwu Hu

**Affiliations:** 1 State Key Laboratory of Stress Biology for Arid Areas, Northwest A&F University, Xianyang 712100, China; lvjy890727@sdu.edu.cn (J.L.); guoyuan2109@163.com (Y.G.); dcl220117@163.com (C.D.); yuhb202208@163.com (H.Y.); guoli@nwafu.edu.cn (L.G.); s786429264@163.com (L.L.); hxzhao212@nwsuaf.edu.cn (H.Z.); 2 Oil Crops Research Institute of the Chinese Academy of Agricultural Sciences, Key Laboratory of Biology and Genetic Improvement of Oil Crops, Ministry of Agriculture and Rural Affairs, Wuhan 430062, China

**Keywords:** rapeseed (*Brassica napus* L.), *BnERF114.A1*, plant architecture, shoot branching, apical dominance, seed yield

## Abstract

Plant architecture is crucial for rapeseed breeding. Here, we demonstrate the involvement of *BnERF114.A1*, a transcription factor for ETHYLENE RESPONSE FACTOR (ERF), in the regulation of plant architecture in *Brassica napus*. *BnERF114.A1* is a member of the ERF family group X-a, encoding a putative 252-amino acid (aa) protein, which harbours the AP2/ERF domain and the conserved CMX-1 motif. *BnERF114.A1* is localised to the nucleus and presents transcriptional activity, with the functional region located at 142–252 aa of the C-terminus. GUS staining revealed high *BnERF114.A1* expression in leaf primordia, shoot apical meristem, leaf marginal meristem, and reproductive organs. Ectopic *BnERF114.A1* expression in *Arabidopsis* reduced plant height, increased branch and silique number per plant, and improved seed yield per plant. Furthermore, in *Arabidopsis*, *BnERF114.A1* overexpression inhibited indole-3-acetic acid (IAA) efflux, thus promoting auxin accumulation in the apex and arresting apical dominance. Therefore, *BnERF114.A1* probably plays an important role in auxin-dependent plant architecture regulation.

## 1. Introduction

Since the “Green Revolution” of wheat and rice in the 1960s, which substantially increased crop yield, much attention has been paid to the improvement of plant architecture for enhancing the seed yield of rapeseed (canola, *Brassica napus* L.). Plant architecture is the three-dimensional organisation of plant organs, enabling the plant to adapt to and survive in diverse environments. Plant architecture affects crop yield [1]. Therefore, crop breeders have focused on aerial architecture, including plant height, branch or tiller number and angle, leaf shape, size, and angle, and inflorescence morphology [2,3,4]. Rapeseed is the most widely distributed and cultivated oil crop in China, providing over 55% of edible oil in daily life. With the rapid development of the national economy and transfer of a large volume of the rural labour force to urban areas, agricultural farming methods warrant urgent revision. In this light, simplification and mechanisation have become the major targets of rapeseed breeding and industrial development in China [5]. The ideal rapeseed plant architectural traits for mechanical harvest are dwarfness, lodging resistance, branch habit suitable for dense plantation, uniform ripening, cracking resistance, and early maturation [6,7]. Traits related to the branch habit of rapeseed plants include branch position, angle, and number, which are important in the breeding of varieties suitable for mechanised farming [8]. Therefore, the knowledge of rapeseed branch traits and their genetic regulatory mechanisms is fundamental to the genetic improvement of varieties for mechanical harvest.

In vascular plants, the main stem is derived from the primary shoot apical meristem (SAM), and branches differentiate from the axillary meristem (AM) [9]. Branching occurs via a two-step process: initiation of AM in each leaf axil to form a bud, and subsequent outgrowth of the bud [10]. In some plants, the axillary bud is dormant as a result of complex interactions between endogenous developmental signals, including auxin, cytokinin (CK), and strigolactones, and environmental factors, such as shade [11,12,13].

Plant architecture, represented by a set of genetically controlled agronomic traits, is closely linked to plant hormones and signal transduction. The Green Revolution of rice and wheat has primarily been attributed to reduction in plant height through the disruption of gibberellin synthesis (*sd1*) and inhibition of DELLA protein (*Rht-B1b* and *Rht-D1b*) degradation [14,15]. Indole-3-acetic acid (IAA), the first-discovered plant hormone, plays pivotal roles in the regulation of plant architecture. Apical dominance is driven by IAA accumulation in the apex. As an antagonistic hormone of IAA, CK suppresses apical dominance. *Isopentenyltransferase* (*IPT*), a CK biosynthetic gene, promotes the outgrowth of axillary buds and inhibits auxin-dependent apical dominance [16].

The phytohormone auxin, which is predominantly represented by IAA, regulates multiple plant developmental processes, such as embryo development, organofaction, lateral root emergence, tropic growth, vascular tissue differentiation, apical dominance and lateral bud initiation through its differential distribution within plant tissues [17,18]. This differential distribution pattern is established by auxin biosynthesis and transport. The key enzymes involved in the main auxin biosynthetic pathways belong to the YUC family of flavin monooxygenases, which consists of 11 *YUC* genes in *Arabidopsis* [19]. Differential distribution of synthesised auxin is mainly achieved by a polar cell-to-cell transport system. Auxins are transported to different parts of the plant mainly by four auxin-transporting families, including AUX/LAX (auxin influx carriers), PIN (PIN-FORMED, auxin efflux carriers), ABCB (ATP-binding cassette B (ABCB)/P-glycoprotein (PGP), and PILS (PIN-Likes). PIN and AUX/LAX are distributed in a polar fashion in the plasma membrane, whereas PGP and PILS are present in a non-polar fashion [18,20,21,22,23]. With the exception of AUX/LAX, auxin transporters harbour N-and C-terminal conserved domains along with a variable hydrophilic loop in the transmembrane domain [18,20,21,22]. The functional diversity of different transporters might base on different phosphorylated sites in a variable hydrophilic loop [18,20,21]. The PIN, AUX/LAX, and PGP transporters mediate long distance auxin transport, whereas PILS and PIN5 proteins are involved in intracellular auxin homeostasis [18].

APETALA2/ETHYLENE RESPONSE FACTOR (AP2/ERF) is one of the largest and most important plant-specific transcription factor superfamilies, represented by 147 members in *Arabidopsis* [24]. All members of this superfamily are characterised by the presence of one or two functional 68-amino acid (aa) repeat motifs, termed the AP2 domains, which possess DNA-binding ability [25,26,27]. Based on their structural features, the AP2/ERF family members in *Arabidopsis* are classified into four subfamilies: AP2 (18 members), RAV (6 members), EREB-DREB (122 members), and AP2P-ISAMDD1 (1 member) [24]. The AP2 subfamily, characterised by the presence of two AP2 domains, includes several key regulators of different plant growth and developmental processes. The RAV-type transcription factors harbour a single AP2 domain and another conserved C-terminal DNA-binding domain called B3, which responds to touch-related stimuli [28,29]. The largest subfamily EREB-DREB is further divided into 12 subgroups, namely I to X, VI-L, and Xb-L. The members of the X subgroup (ERF108 to ERF115) possess a hallmark conserved N-terminal sequence and are involved in wound signalling and tissue repair [24,27].

The *Arabidopsis* AP2/ERF transcription factor gene *ERF BUD ENHANCER* (*EBE*) (*At5g61890*, also known as *AtERF114*), which is highly expressed in proliferating tissues, regulates cell proliferation, axillary bud outgrowth, and shoot branching [30]. A recent study revealed that *ERF115*, an AP2/ERF transcription factor of the X subgroup, is involved in wound-induced stem cell division and cell sensitivity to auxin [31]. In addition, *ERF115* functions as a repressor of adventitious root initiation by integrating the crosstalk between jasmonic acid (JA) and CK [32]. *ERF109*, another X subgroup member, is induced by wounding, and promotes auxin biosynthesis [33]. In a previous study, we observed that monosulphuron ester sodium (MES) could act as a novel chemical hybridisation agent for rapeseed. Specifically, even at a low concentration (10 mL per plant of 0.1 μg·mL^−1^), MES induced male sterility and increased branch number in rapeseed [34,35]. Comparative transcriptomics between MES-treated and control plants revealed that approximately 36% (542/1501) of the differentially expressed transcripts associated with anther development, including certain kinases and various transcription factors, were significantly upregulated. Amongst these, an integrase-type DNA-binding superfamily protein was found to share high homology with *AtERF114* [35], and was therefore named *BnERF114*. *AtERF114* belongs to the X subgroup of the ERF transcription factor family, and harbours the AP2/ERF domain, which may regulate cell proliferation and promote axillary bud generation and branch growth [30]. However, the characteristics and biological functions of *BnERF114* in rapeseed (*Brassica napus*) remain unclear, and whether the phenotype with increased branch number is related to *BnERF114* upregulation in rapeseed plants treated with a low concentration of MES warrants further investigation.

Therefore, to elucidate the biological roles of *BnERF114* in plant development, *BnERF114* and its paralogs were isolated and subjected to phylogenetic analysis in the present study. The molecular characteristics of *BnERF114* were analysed and the biological function of ectopic expression of *BnERF114.A1* in *Arabidopsis* was explored. Our results suggest *BnERF114.A1* probably regulates plant shoot branching and plant architecture through an auxin-dependent way in *Arabidopsis*.

## 2. Results

### 2.1. BnERF114s Are Orthologues of AtERF114

**Figure 1:**
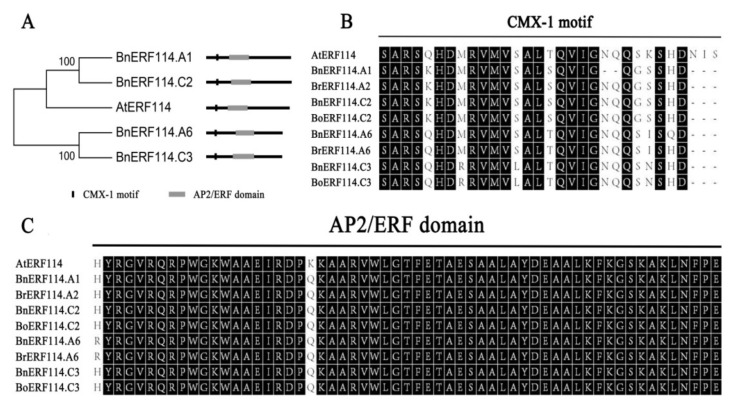
The conserved structures of ERF114 proteins. (**A**) phylogenetic analysis and the structure diagrams of ERF114 proteins in *Brassica napus* and *Arabidopsis*. (**B**) alignment of the CMX-1 motif of ERF114 in *B. napus*, *B. rapa*, and *B. oleracea.* (**C**) alignment of the conserved AP2/ERF domain of ERF114 in *B. napus*, *B. rapa*, and *B. oleracea*. In (**B**,**C**), amino acids with black background indicate conserved amino acid residues across ERF114s.

To clone the orthologues of *EBE* (*AtERF114*) in *B. napus*, we blasted the full-length protein sequence of AtERF114 (AT5G61890.1) against the *Brassica* non-redundant protein sequence database of NCBI. As a result, eight highly homologous genes were identified: four from *B. napus* and two each from *B. rapa* and *B. oleracea* (Table S2). To identify the evolutionary relationships of *BnERF114s* in *B. napus*, phylogenetic and homologous analyses were conducted using the AP2/ERF conserved domain of BnERF114s, BrERF114s, BoERF114s, and *Arabidopsis* AP2/ERF superfamily genes. The result suggested that BnERF114.C2 is derived from BoERF114.C2, BnERF114.A6 from BrERF114.A6, and BnERF114.C3 from BoERF114.C3. Meanwhile, *BnERF114.A1* may have been derived from BrERF114.A2 (Figure S1A). Phylogenetic analysis of *BnERF114s* and all *Arabidopsis ERF* family members showed that *BnERF114s* are orthologues of *AtERF114* (Figure S1B) and belong to the subgroup X-a of the AP2/ERF superfamily [24]. Consistent with *AtERF114*, all four *BnERF114s* possess two exons and one intron, and the encoding proteins harbour the conserved CMX-1 motif (Figure 1A,B) and an AP2/ERF domain (Figure 1A,C). These results indicate that *BnERF114s* are orthologues of *AtERF114*.

### 2.2. BnERF114.A1 Is Expressed in Proliferating Tissue

**Figure 2:**
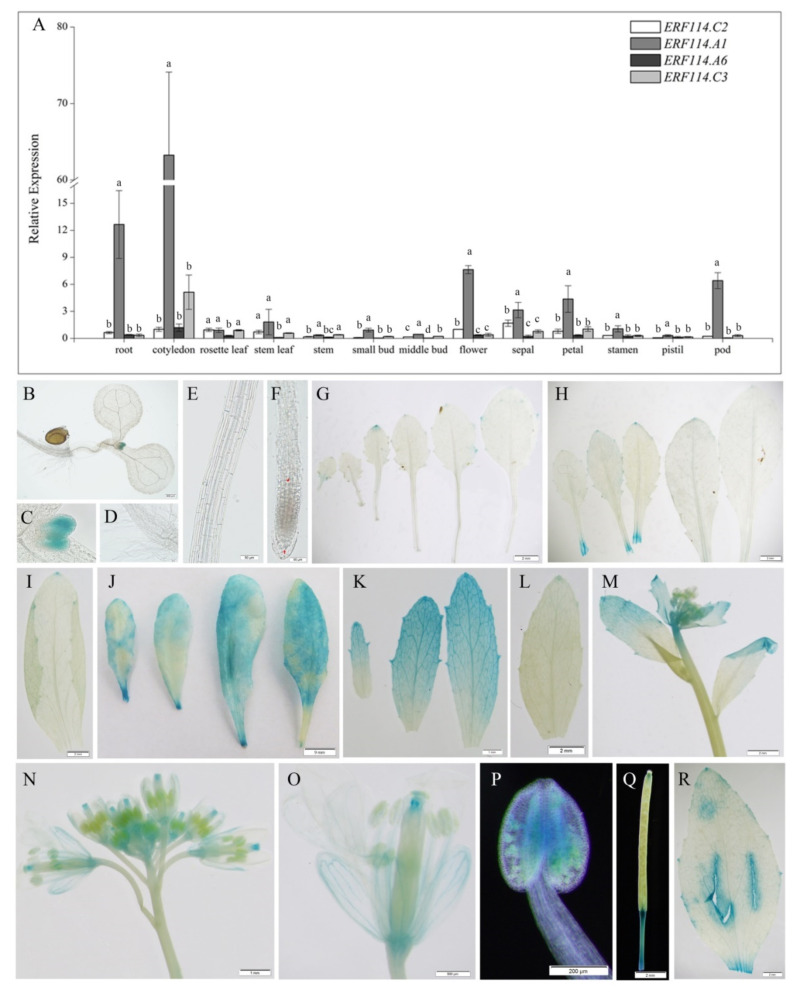
*BnERF114s* expression profile. (**A**) relative expression levels of *BnERF114s* in rapeseed different tissues. Gene expression levels were normalised to the reference gene *BnUBC21* (Gene ID: 106348550). Relative expression levels of *BnERF114s* were compared with the expression of *BnERF114.C2* in flower. Each column represents the mean of three independent biological replicates, and the error bars indicate standard deviation. Different lowercase letter means significant difference at *p* = 0.05 level. (**B**) to (**R**), *BnERF114.A1* expression patterns identified through histochemical GUS staining in *Arabidopsis* OE_114_-46-3 transgenic line. (**B**) cotyledon (bar = 100 μm). (**C**) leaf primordium (bar = 20 μm). (**D**) root hair (bar = 50 μm). (**E**) root elongation zone (bar = 50 μm). (**F**) root tip (bar = 50 μm). (**G**–**J**) the youngest to the oldest rosette leaf, respectively (**G**–**I**, bars = 2 mm; **J**, bar = 9 mm). (**K**) new cauline leaves (bar = 1 mm). (**L**) mature cauline leaf (bar = 2 mm). (**M**) main inflorescence (bar = 2 mm). (**N**) branched inflorescence (bar = 1 mm). (**O**) flower (bar = 500 μm). (**P**) anther (bar = 200 μm). (**Q**) pot (bar = 2 mm). (**R**) wounded mature rosette leaf (bar = 2 mm).

To explore the spatiotemporal expression profile of *BnERF114s*, we measured their transcriptional levels in 13 different tissues of the rapeseed cultivar ZS9. The expression level of *BnERF114.A1* was higher than that of the other three genes (*BnERF114.C2*, *BnERF114.A6*, and *BnERF114.C3*) in all tissues, indicating that *BnERF114.A1* probably worked as a major gene amongst the four *BnERF114s*. *BnERF114.A1* exhibited higher expression levels in roots, cotyledons, flowers, sepals, petals, and pods, but lower expression levels in rosette leaves, stem, small and middle buds, stamens, and pistils (Figure 2A). Furthermore, we tested *BnERF114.A1* expression levels in the shoot apex of rapeseed ZS9. The result showed that the *BnERF114.A1* expression level in the shoot apex was five times higher compared with that in the cauline leaf (Figure S2). Considering the expression profiles of *BnERF114s* during rapeseed development, *BnERF114.A1* was selected for subsequent functional analyses.

To further detect the expression profile of *BnERF114.A1*, the native *BnERF114.A1* promoter (114pro), including 1836 bp upstream of ATG and the CDS of *BnERF114.A1,* were cloned into a binary expression vector pCAMBIA3301. The recombinant construct *114pro::BnERF114.A1-GUS* was introduced into *Arabidopsis* using *Agrobacterium tumefaciens*-mediated transformation, and GUS activity in different tissues of transgenic *Arabidopsis* plants was analysed. *BnERF114.A1* was markedly expressed in leaf primordia, shoot apical meristem, leaf marginal meristem, young cauline leaves (Figure 2B–I), senescent leaves, cutting positions (Figure 2J), and reproductive organs, including pistils and anthers (Figure 2K–Q). Therefore, mechanical injuries were inflicted on middle-aged leaves 45 min before GUS staining. Mechanical injury could strongly and rapidly induce *BnERF114.A1* expression (Figure 2R). These results suggest that *BnERF114.A1* is involved in cell proliferation and wound response. However, the expression profiles of *BnERF114.A1* shown by GUS staining were not exactly the same as those identified by qPCR in some tissues, such as roots and cotyledons (Figure 2A,B,E,F). This might be caused by the heterogeneous expression of *BnERF114.A1* in *Arabidopsis*.

### 2.3. BnERF114.A1 Is Mainly Localised to the Nucleus and Exhibits Transcriptional Activity

**Figure 3:**
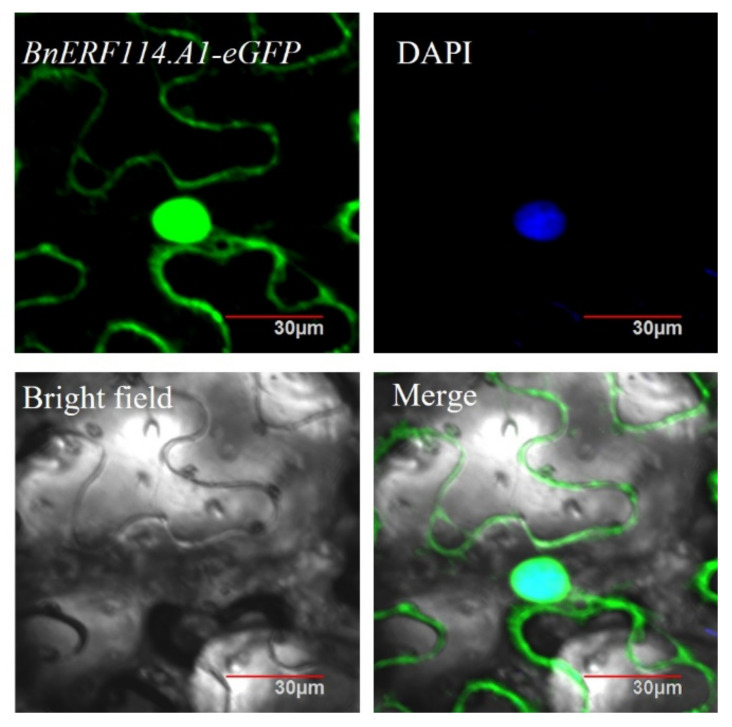
Subcellular localisation of *BnERF114.A1*. Transient expression vector *35S::BnERF114.A1-eGFP* was introduced into *Nicotiana benthamiana* leaf cells. The infected leaf was stained using DAPI as nuclear localisation marker. Bars = 30 μm.

To confirm the subcellular localisation of *BnERF114.A1*, the full-length CDS of *BnERF114.A1* from the rapeseed cultivar ZS9 was isolated and fused with *eGFP* in frame to construct the transient expression vector *p35S::BnERF114.A1-eGFP*. This transient expression vector was introduced into *Nicotiana benthamiana* leaf cells to express the BnERF114.A1-eGFP fusion protein, with DAPI as nuclear localisation marker. The results indicated that *BnERF114.A1* was mainly localised to the nucleus, while relatively weak signals could be observed in plasma membrane (Figure 3).

**Figure 4:**
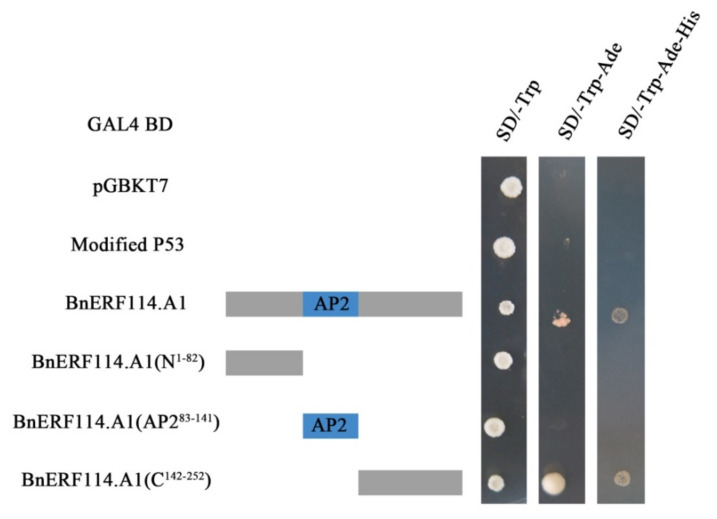
Transcriptional activity and functional region of *BnERF114.A1* tested by yeast system. GAL4 BD indicates that Modified P53 (a modified P53 protein only containing DNA binding domain), intact *BnERF114.A1*, and truncated *BnERF114.A1* were separately fused with GAL4 BD. pGBKT7 is empty vector used as a control. Modified P53 is used as a negative control.

The AP2 domain of *BnERF114.A1* was predicted to be 83–141 amino acid. According to a previous report [24], AP2 is a DNA-binding domain. Therefore, to examine the transcriptional activity of *BnERF114.A1* and functional domain position, we introduced different fragments of *BnEFR114.A1* CDS, including full-length CDS, N-terminus fragment (246-), AP2 domain (-177-), and C-terminus fragment (-333) into the pGBKT7 vector. The recombinant constructs were transformed into yeast *AH109* and screened on yeast medium SD/-Trp and SD/-Trp/-Ade/-His media. Yeast self-activation experiments confirmed that *BnERF114.A1* exhibited transcriptional activity, and the functional region was located at 142–252 aa of the C-terminus (Figure 4).

### 2.4. Ectopic BnERF114.A1 Expression in Arabidopsis Reduced Plant Height and Increased Branch Number

**Figure 5:**
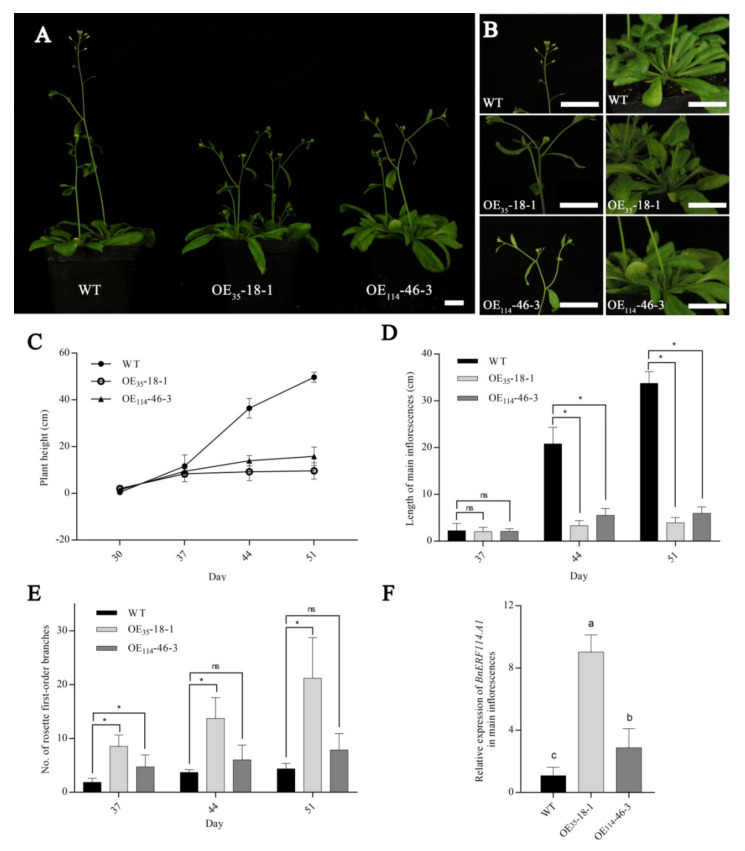
Effects of *BnERF114.A1* ectopic expression on plant phenotype. WT, wild-type (Col-0) *Arabidopsis*; OE_35_-18-1, transgenic line expressing *BnERF114.A1-eGFP-GUS* under the 35S promoter; OE_114_-46-3, transgenic line expressing *BnERF114.A1-GUS* under the 114pro promoter. (**A**) phenotypes of OE_35_-18-1 and OE_114_-46-3 transgenic plants at 40 days old. (**B**) phenotypes of the main inflorescence and first rosette branches of OE_35_-18-1 and OE_114_-46-3 transgenic plants at 40 days old, in (**A**,**B**) Bars = 2 cm. (**C**) plant height of OE_35_-18-1 and OE_114_-46-3 transgenic lines at 30, 37, 44, and 51 days, each point represents mean ± SD of 5–10 independent individuals. (**D**) length of the main inflorescence in OE_35_-18-1 and OE_114_-46-3 transgenic lines at 37, 44, and 51 days; each column represents mean ± SD of 5–10 independent individuals. (**E**) number of first rosette branches in OE_35_-18-1 and OE_114_-46-3 transgenic lines at 37, 44, and 51 days. In (**D**,**E**), asterisks indicate significant differences at the *p* = 0.05 level, and “ns” indicates non-significant differences at the *p* = 0.05 level. (**F**) relative *BnERF114.A1* expression in the main inflorescence of OE_35_-18-1 and OE_114_-46-3 plants at 40 days old; gene expression levels were normalised to the reference gene *AtUBC21* (*At5g25760*); each column represents mean ± SD from three independent biological replicates, and each biological replicate included five individuals. Different lowercase letters indicate significant differences at the *p* = 0.05 level.

To explore the effects of *BnERF114.A1* on plant architecture, we developed more than ten *35S::BnERF114.A1* (OE_35_), *114pro::BnERF114.A1* (OE_114_), *35S::GUS,* and *114pro::GUS Arabidopsis* transgenic lines, respectively. Because of the strong ectopic expression lines performing a sterile phenotype, we used relatively low-expression transgenic lines for functional analysis. Before the beginning of the bolting stage (-30 days after planting), no phenotypic differences were detected between the wild-type (Col-0) and transgenic plants ectopically expressing *BnERF114.A1*. At 40 days after planting, ectopic *BnERF114.A1* expression significantly inhibited the elongation of the main inflorescence, thus reducing plant height (here, OE_35_-18-1 as the representative of the OE_35_ lines and OE_114_-46-3 as the representative of the OE_114_ lines showed similar phenotypes within the same genotype) (Figure 5A–D). In addition, ectopic *BnERF114.A1* expression accelerated the emergence and outgrowth of lateral branches and first-order rosette branches in transgenic lines (Figure 5E). After 37 days of growth, the OE_35_ transgenic plants produced more rosette branches than the wild-type plants (Figure 5E). Further, the expression levels of *BnERF114.A1* in transgenic and wild-type plants were determined. Both transgenic lines (OE_35_ and OE_114_) showed significantly higher expression levels of *BnERF114.A1* in the main inflorescences (Figure 5F). The phenotype of *Arabidopsis* transgenic lines of both empty vectors (*35S::GUS, 114pro::GUS*) performed no differently to that of wild-type plants (Data not shown here). These results suggest that *BnERF114.A1* is involved in the regulation of plant branch habit and apical dominance.

### 2.5. BnERF114.A1 Overexpression in Arabidopsis Increased Seed Yield

**Figure 6:**
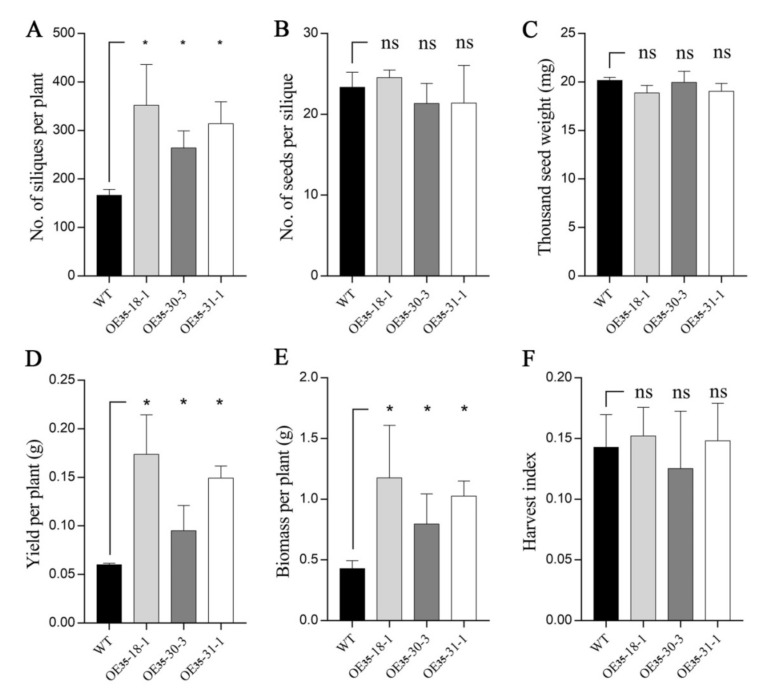
Yield-related traits of *BnERF114.A1* transgenic plants. (**A**) number of siliques per plant. (**B**) number of seeds per silique. (**C**) thousand-seed weight (mg). (**D**) yield per plant (g). (**E**) biomass per plant (g). (**F**) harvest index. Values represent the data of 3 wild-type and 10 OE_35_ transgenic plants. Asterisk indicates significant differences at the *p* = 0.05 level. “ns” indicates non-significant differences at the *p* = 0.05 level.

To evaluate the effects of *BnERF114.A1* on seed yield, we investigated the yield-related traits of wild-type and transgenic *Arabidopsis* plants overexpressing *BnERF114.A1*. Since the OE_35_ lines showed significantly higher expression levels of *BnERF114.A1* than the OE_114_ lines, we compared the yield-related traits between the OE_35_ transgenic and wild-type plants. The OE_35_ transgenic plants produced more siliques per plant (n = 270–320) than the wild-type plants (n = 170) (Figure 6A), although the number of seeds per silique and thousand-seed weight were similar between the two groups (Figure 6B,C). Moreover, the OE_35_ transgenic plants showed a much higher seed yield (0.12–0.22 g) and biomass (0.8–1.2 g) per plant than the wild-type plants (0.06 g and 0.5 g, respectively) (Figure 6D,E). These results may be because ectopic *BnERF114.A1* expression potently promoted the emergence and outgrowth of shoot branches (Figure 5E), which increased the number of siliques per plant (Figure 6A).

### 2.6. Ectopic BnERF114.A1 Expression Affected IAA Efflux in the Main Inflorescence of Arabidopsis

**Figure 7:**
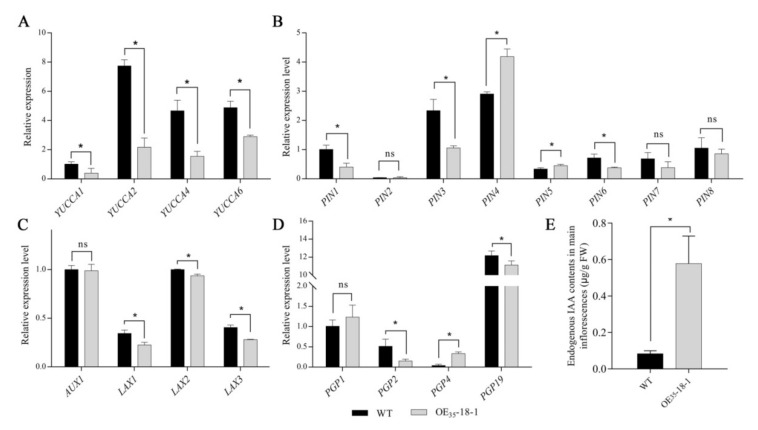
Relative expression levels of IAA-related genes and endogenous IAA level in main inflorescence of transgenic plants. (**A**) relative expression levels of four *YUCCA* genes (*AtYUCCA1* [*At4g32540*], *AtYUCCA2* [*At4g13260*], *AtYUCCA4* [*At5g11320*], and *AtYUCCA6* [*At5g25620*]); the expression level *AtYUCCA1* in the wild-type (WT) being set as a unit. (**B**) relative expression levels of eight *PIN* family genes (*AtPIN1* [*At1g73590*], *AtPIN2* [*At5g57090*], *AtPIN3* [*At1g70940*], *AtPIN4* [*At2g01420*], *AtPIN5* [*At5g16530*], *AtPIN6* [*At1g77110*], *AtPIN7* [*At1g23080*], and *AtPIN8* [*At5g15100*]); the expression level of *AtPIN1* in the WT being set a unit. (**C**) relative expression level of four *AUX/LAX* family genes (*AtAUX1* [*At2g38120*], *AtLAX1* [*At5g01240*], *AtLAX2* [*At2g21050*], and *AtLAX3* [*At1g77690*]), and the expression level of *AtLAX2* in the WT being set as a unit. (**D**) relative expression levels of four *PGP* family genes (*AtPGP1* [*At2g36910*], *AtPGP2* [*At4g25960*], *AtPGP4* [*At2g47000*], and *AtPGP19* [*At3g28860*]); the expression level of *AtPGP1* in the WT being set as a unit. Gene expression levels were normalised to the reference gene *AtUBC21* (*At5g25760*). (**E**) endogenous IAA levels in the main inflorescences of the WT and transgenic plants at 40 days old. Each column represents mean ± SD from three independent biological replicates, and each biological replicate included five individuals. Asterisk indicates significant differences at the *p* = 0.05 level; “ns” indicates non-significant differences at the *p* = 0.05 level.

In plants, apical dominance is linked to the synthesis and distribution of auxin. Auxin synthesised from the apex of the plant is transported in a polar manner down to the lateral branches, thus inhibiting the growth of the lateral buds. Decapitation of *Arabidopsis* induced *AtERF114* expression in the five uppermost lateral branches, in addition to the main inflorescences; thus, these plants produced more lateral branches and exhibited cespitose phenotypes [30]. In the present study, ectopic *BnERF114.A1* expression inhibited apical dominance in *Arabidopsis*; therefore, we hypothesised that either auxin synthesis and/or auxin polar transport was blocked. To test our hypothesis, we examined the expression levels of the IAA biosynthetic genes *YUCCA1*, *YUCCA2*, *YUCCA4*, and *YUCCA6*, which play pivotal roles in auxin-dependent apex dominance [36]. *YUCCA1*, *YUCCA2*, *YUCCA4*, and *YUCCA6* expression levels in the transgenic plants were significantly decreased compared with those in the wild-type plants (Figure 7A). We further examined the expression levels of the IAA transporters *PINs*, *AUX/LAXs*, and *PGPs*, in the main inflorescences of transgenic and wild-type *Arabidopsis* plants. The expression levels of *PIN1*, *PIN3*, *PIN6*, *LAXs*, *PGP2*, and *PGP19* were significantly decreased to various degrees (Figure 7B–D), but the expression levels of *PIN4*, *PIN5*, and *PGP4* were increased in the transgenic plants. These results suggest that ectopic *BnERF114.A1* expression inhibits both auxin synthesis and flux in *Arabidopsis*. Therefore, we speculated that IAA was accumulated in the main inflorescences and cauline leaves of transgenic plants. To confirm this, endogenous IAA levels in the main inflorescences of wild-type and OE_35_-18-1 transgenic plants were measured through liquid chromatography. As expected, the IAA content of the main inflorescences of the transgenic plants was markedly increased compared with that of the wild-type plants (Figure 7E). However, the IAA content of cauline leaves was lower in the OE_35_-18-1 transgenic plants than in the wild-type plants (Figure S3). These results indicate that ectopic *BnERF114.A1* expression affects endogenous levels of IAA in the main inflorescences and the flux of IAA in *Arabidopsis*.

## 3. Discussion

Ideal plant architecture (ideotype) is an important objective of rapeseed breeding [7]. In this context, the identification and characterisation of various candidate genes regulating the ideotype will contribute to the molecular breeding for this crop. In the present study, we characterised a novel candidate ERF transcription factor gene, *BnERF114.A1*, which belongs to the subgroup X-a of the ERF superfamily. Our results indicated that *BnERF114.A1* is localised to the nucleus and possesses transcriptional activity. Moreover, *BnERF114.A1* is expressed in proliferating tissues. Ectopic *BnERF114.A1* expression in *Arabidopsis* reduced plant height, inhibited apical dominance, increased shoot branch number, and improved seed yield and biomass per plant. However, this did not affect the harvest index. Finally, *BnERF114.A1* regulated plant architecture by affecting the efflux and endogenous levels of IAA in the main inflorescences of *Arabidopsis*.

A previous study demonstrated high *AtERF114* expression in undifferentiated suspension culture cells and callus [37]. Furthermore, it was confirmed that *AtERF114* expression was elevated at the S phase of the cell cycle, suggesting that *ERF114* regulates cell proliferating and division [38]. In *Arabidopsis*, *AtERF114* is prominently expressed in root tips, shoot apices, young leaves, and reproductive organs [30]. In the present study, *BnERF114.A1* was consistently expressed in actively proliferating tissues, such as leaf primordia, shoot apical meristem, leaf marginal meristem, young cauline leaves, and reproductive organs (Figure 4). In addition, previous studies have shown that *ERF114* and its homologues, such as *ERF109/ERF115*, could be induced by reactive oxygen species (ROS)-mediated signalling induced by stress or wounding [31,39,40]. Consistently, in the present study, strong GUS activity was detected in senescent and wounded leaves (Figure 2J,R), suggesting that *BnERF114.A1* also responds to ROS-mediated signalling.

*AtERF114* promotes axillary bud formation and outgrowth [30]. In the present study, *BnERF114.A1* overexpression in *Arabidopsis* remarkably inhibited apical dominance and promoted first-order lateral branch formation (Figure 5), indicating that *BnERF114.A1* probably serves similar functions to *AtERF114* in promoting the emergence and outgrowth of axillary buds. *AtERF114* belongs to the X-a subgroup of the ERF transcription factor superfamily. This group comprises eight members, namely ERF108–ERF115 [24]. Wound-induced JA can activate ERF109, which upregulates the expression of a tryptophan biosynthesis-related gene (*ASA1*) in the auxin biosynthetic pathway, resulting in *de novo* root regeneration [41]. In this context, we measured auxin levels in *BnERF114.A1* transgenic lines and observed that *BnERF114.A1* overexpression markedly enhanced auxin accumulation in the main inflorescence, suggesting that *BnERF114.A1* produces a similar effect on auxin accumulation as *AtERF109*.

Auxin plays vital roles in regulating plant shoot branching and maintaining apical dominance [42]. The long-distance transport of endogenous IAA occurs in a polar manner, depending on the auxin polar transport carriers [18]. The auxin efflux carrier PIN1 is expressed in vascular tissues and root primordia, regulating adventitious root development and floral bud formation [43,44]. In rice, *OsPIN1* downregulation increased plant tiller number depending on the auxin content [45]. Moreover, the intercellular auxin transporter PGP19 is colocalised with PIN1, and can stabilise PIN1 on the cytomembrane, synergistically regulating auxin long-distance basipetal transport [46,47,48]. The triple mutant *pin3 pin4 pin7* partially rescues the branching phenotype caused by *arr1* mutant, indicating that the positive effects of *PIN3*, *PIN4* and *PIN7* on shoot branching are very important [49]. In the present study, ectopic *BnERF114.A1* expression suppressed *PIN1*, *PIN3*, *PIN6*, *LAXs*, *PGP2*, and *PGP19* expression, but increased *PIN4*, *PIN5*, and *PGP4* expression (Figure 7B–D). The interplay of the different auxin transporters finally resulted in the block of IAA efflux from the apex to the axillary bud. It has been reported that *PIN5* is involved in intracellular auxin homeostasis, but not in long-distance auxin transport [18]. However, the relative expression of *PIN3* decreased significantly compared with wild-type plants, while the expression of *PIN7* made no difference in this study. Thus, we speculated that the increased expression of *PIN4* and *PIN5* was probably a response to the arrested auxin efflux. In previous studies, the directionality of PGP4 has been shown to be regulated by the concentration of auxin. PGP4 can act as an auxin influx carrier under low IAA concentration, and as an auxin efflux carrier under high IAA concentration [50]. In the present study, the relative expression of *PGP4* displayed a significant increase in transgenic plants. This might be caused by the markedly increased IAA levels in the main inflorescence of transgenic plants (Figure 7E). Finally, the inhibition of IAA efflux and accumulation of IAA in the main inflorescence reduced plant height and increased branch number (Figure 5A).

YUCCAs play important roles in endogenous IAA biosynthesis. IAA synthesis-deficient double mutants *yuc1yuc4* and *yuc2yuc6*, triple mutants *yuc1yuc2yuc6*, *yuc1yuc2yuc4*, and *yuc1yuc4yuc6*, and quadruple mutant *yuc1yuc2yuc4yuc6* exhibited markedly reduced plant height and suppressed apical dominance [36]. In the present study, ectopic *BnERF114.A1* expression suppressed *YUCCA1*, *YUCCA2*, *YUCCA4*, and *YUCCA6* expression (Figure 7A), but increased IAA levels in the main inflorescence (Figure 7E). Therefore, we speculated that this increase in IAA levels might be the result of IAA accumulation upon the blockade of IAA efflux from the apex to the axillary bud. However, decreased IAA levels in cauline leaves indicated the arrest of IAA synthesis and transport (Figure S3). The suppression of *YUCCA* expression may be the result of feedback inhibition via IAA accumulation. This also explains that the blockade of the elongation of transgenic plant main stems arises approximately 10 days after bolting, rather than at the beginning of the bolting stage.

In conclusion, we isolated and characterised *BnERF114.A1*, an orthologue of *AtERF114* in *Brassica napus*. Ectopic *BnERF114.A1* expression in *Arabidopsis* reduced plant height, increased shoot branch number, arrested apical dominance, and improved seed yield per plant. Furthermore, ectopic *BnERF114.A1* expression regulated plant architecture through the blockade of auxin efflux from the apex to the base. Therefore, *BnERF114.A1* probably plays an important role in plant architecture regulation in an auxin-dependent way in *Arabidopsis*.

## 4. Materials and Methods

### 4.1. Plant Material and Growth Condition

*Brassica napus* ‘Zhongshuang No.9′ (ZS9) was planted in the Yangling Regional Test Station of Crop Varieties, Shaanxi, China (34.29° N, 108.06° E) during the growing season of 2017–2018. This cultivar was introduced from the Oil Crops Research Institute of the Chinese Academy of Agricultural Sciences, Wuhan, China, and selfed for at least 10 generations prior to use in the present experiment. *Arabidopsis thaliana* (Col-0) and transgenic plants were grown at 22 °C under a 16-h light/8-h dark cycle (light intensity 6000–9000 lx) and ~60% humidity in a phytotron.

### 4.2. Gene Characterisation and Phylogenetic Analysis

The protein sequence of AtERF114 (AT5G61890.1) was obtained from the TAIR (17 November 2018, http://www.arabidopsis.org/) website, and it was used as the query sequence to search for homologous genes in *Brassica* using the PSI-BLAST tool of NCBI (18 November 2018, http://blast.ncbi.nlm.nih.gov/Blast.cgi). To confirm the genetic relationships of ERF114s from *Brassica* and *Arabidopsis*, phylogenetic analysis was performed using MEGA 5 on the conserved AP2 domains (SMART accession number: SM00380). Moreover, sequences of 122 AP2/ERF family proteins from *Arabidopsis* were downloaded from the TAIR website and used to construct a neighbour-joining (NJ) tree [24].

### 4.3. Nucleic Acid Isolation

Total RNA was extracted from rapeseed different tissues and transgenic *Arabidopsis* using a commercial extraction kit (E.Z.N.A.^®^ Plant RNA Kit, OMEGA, Norcross, GA, USA) following the manufacturer’s protocol, and digested with RNA-free DNase I (Invitrogen) to remove DNA contamination. Genomic DNA was extracted from young leaf samples of rapeseed and *Arabidopsis* plants using the cetyltrimethylammonium bromide (CTAB) method [51]. The quality of the isolated RNA and DNA samples was assessed using 2.0% and 0.8% agarose gel electrophoresis, respectively.

### 4.4. Cloning of the Coding Sequence (CDS) and Promoter of BnERF114.A1 in Rapeseed

For cloning, first-strand cDNA was synthesised using total RNA isolated from the young pods of rapeseed ZS9 using the GoScript™ Reverse Transcription System (Promega, Madison, WI, USA) according to the manufacturer’s protocol. The CDS of *BnERF114.A1* was isolated from cDNA through polymerase chain reaction (PCR) using the high-fidelity thermostable DNA polymerase KOD-FX-NEO (TOYOBO) and the primer pair BnERF114.A1-F and BnERF114.A1-R (Table S1). The PCR conditions were as follows: pre-denaturation at 98 °C for 2 min, followed by 40 cycles of 98 °C for 10 s, 58 °C for 30 s, and 68 °C for 1 min, and final extension at 68 °C for 7 min. The PCR products were cloned into the pMD19-T vector (TaKaRa, Dalian, China), and five randomly selected clones were sequenced.

The promoter of *BnERF114.A1* was cloned from the genomic DNA of ZS9 using the primer pair 114pro-F and 114pro-R (Table S1). The PCR conditions were as follows: pre-denaturation at 98 °C for 2 min, followed by 40 cycles at 98 °C for 10 s, 60 °C for 30 s, and 68 °C for 1 min, and final extension at 68 °C for 7 min. The PCR products were cloned into the pMD19-T vector (TaKaRa, Japan), and five randomly selected clones were sequenced.

### 4.5. Subcellular Localisation of BnERF114.A1

To investigate the subcellular localisation of BnERF114.A1, the primer pair 114SL-F and 114SL-R (Table S1) was used to clone the CDS of *BnERF114.A1*, and a pGreen-*35S::BnERF114.A1-eGFP* expression vector was constructed using T4 DNA ligase (TaKaRa, Japan) at 16 °C for 1 h following EcoRI and SpeI double digestion. The constructed vector was confirmed using restriction analysis and sequencing. The recombinant vector was transformed into *Agrobacterium tumefaciens* GV3101 strain. Agrobacterial suspension containing pGreen-*35S::BnERF114.A1-eGFP* was injected into the lower epidermis of *Nicotiana benthamiana* leaves. The transfected plants were kept in the 16 h light/8 h dark greenhouse for two days at 22 °C. The tobacco leaves were cut into pieces and put into DAPI staining solution (Beyotime, C1005) for 5 min at room temperature. The stained leaf pieces were rinsed with PBST [10 mM PBS (pH = 7.4), 0.1% Tween 20] three times. After treatment with DAPI, the infected leaf pieces were observed under a laser confocal microscope (Zeiss, Germany). The excitation wavelengths for eGFP and DAPI were 488 nm and 405 nm, respectively.

### 4.6. Transcription Activity Analysis of BnERF114.A1

To identify the transcriptional activity of *BnERF114.A1* and its precise region, the full-length CDS sequence of *BnERF114.A1*, the 246 bp region at the 5′-end of *BnERF114.A1* that encodes truncated *BnERF114.A1* only containing 1–82 amino acid residues (aa) at the N-terminal (*BnERF114.A1* N^1–82^), the 177 bp region that encodes truncated *BnERF114.A1* only containing AP2 domain in the 83-141aa region (*BnERF114.A1* AP2^83–141^), and the 333 bp region at the 3′-end of *BnERF114.A1* that encodes truncated *BnERF114.A1* only 142–252 aa at the C-terminal (*BnERF114.A1* C^142–252^) were cloned through PCR using the primer pairs E756-F/E756-R, E246-F/E246-R, E177-F/E177-R, and E333-F/E333-R, respectively (Table S1). The target segments were separately introduced into the pGBKT7 (Clontech) vector using T4 DNA ligase (TaKaRa, Japan) at 16 °C for 1 h following NdeI and EcoRI double digestion (TaKaRa, Japan). The recombinant constructs were transformed into yeast strain *AH109* using the PEG-LiCl method [52]; an empty pGBKT7 vector and a modified pGBKT7-p53 (containing only the DNA-binding region) were used as the negative controls. These colonies were screened on SD/-Trp and SD/-Trp/-Ade/-His media.

### 4.7. Construction of a BnERF114.A1 Overexpression Vector and Arabidopsis Transformation

The CDS of *BnERF114.A1* was introduced into a binary expression vector pCAMBIA3301 driven by the *CAMV 35S* promoter (*35S*) through BamHI and SpeI double digestion and T4 DNA ligation (TaKaRa, Japan) to generate the *35S::BnERF114.A1-GUS* vector. The expression cassette *114pro::BnERF114.A1-GUS* was constructed by substituting the *35S* promoter of the *35S::BnERF114.A1-GUS* vector for the native promoter of *BnERF114.A1* (including 1836 bp upstream of ATG) through EcoRI and BamHI double digestion and T4 ligation (TaKaRa, Japan). The resulting vectors *35S::BnERF114.A1-GUS* and *114pro::BnERF114.A1-GUS* were confirmed by sequencing and transformed into the *Agrobacterium tumefaciens* GV3101 strain. The two expression cassettes and two empty vectors (*35S::GUS, 114pro::GUS*) were transformed into *A. thaliana* (Col-0) using *Agrobacterium tumefaciens*-mediated floral-dip method [53]. Transgenic plants were screened by spraying 0.1% glufosinate (BASTA) on seedling leaves, and independent transgenic homozygous lines were obtained by selfing and spraying 0.1% glufosinate on seedling leaves. To assess the effects of *BnERF114.A1* on transgenic plants, wild-type and homozygous transgenic *Arabidopsis* lines were grown in green houses as described above.

### 4.8. GUS Staining Analysis of BnERF114.A1 Promoter Activity

GUS activity was determined as previously described [54] in T_3_ homozygous transgenic plants carrying *114pro::BnERF114.A1-GUS*. Different tissues were incubated in GUS staining solution (50 mM phosphate buffer (pH 7.2), 0.01% Triton X-100, 2 mM K_3_Fe(CN)_6_, 2 mM K_4_[Fe(CN)_6_]·3H_2_O, 10 mM EDTA, and 0.2 mM X-Gluc) for 16 h at 37 °C. Wounded leaves were infiltrated with the staining solution after injury with a knife and incubated at 37 °C for 45 min. Following treatment with increasing concentrations of ethyl alcohol, plant tissues were observed under a stereomicroscope (Olympus SZ61) coupled to a camera (Canon 3000D).

### 4.9. Quantitative Real-Time PCR (qRT-PCR)

qRT-PCR was used to analyse the spatiotemporal expression patterns of *BnERF114s* in *B. napus.* The cDNA products of different tissues were normalised to the housekeeping gene *ubiquitin-conjugating enzyme 21* of *B. napus* (*BnUBC21*, Gene ID: 106348550) as the internal reference. Three biological and three technical replicates were included for each RNA sample/primer combination in qRT-PCR. Four primer pairs, namely BnERF114.A1-qF/BnERF114.A1-qR, BnERF114.C2-qF/BnERF114.C2-qR, BnERF114.A6-qF/BnERF114.A6-qR, and BnERF114.C3-qF/BnERF114.C3-qR, were used for analysing the expression levels of *BnERF114.A1*, *BnERF114.C2*, *BnERF114.A6*, and *BnERF114.C3*, respectively (Table S1).

The cDNA products of the main inflorescences from wild-type and transgenic plants were normalised to *AtUBC21* (TAIR ID: At5g25760) as the reference to determine the expression levels of auxin polar transport-related and IAA biosynthetic genes. Three biological replicates and three technical replicates were included for each RNA sample/primer combination. qRT-PCR was performed using the primer pairs of *AtPIN1*, *AtPIN2*, *AtPIN3*, *AtPIN4*, *AtPIN5*, *AtPIN6*, *AtPIN7*, *AtPIN8*, *AtAUX1*, *AtLAX1*, *AtLAX2*, *AtLAX3*, *AtPGP1*, *AtPGP2*, *AtPGP4*, *AtPGP19*, *AtYUCCA1*, *AtYUCCA2*, *AtYUCCA4*, and *AtYUCCA6*, as shown in Table S1. The PCR conditions were as follows: pre-denaturation at 94 °C for 4 min, followed by 40 cycles at 94 °C for 20 s, 60 °C for 20 s, and 72 °C for 20 s, and finally, a melting curve was determined from 70 °C to 95 °C for 5 s. qRT-PCR was performed using GoTaq^®^ qPCR Master Mix (A6001, Promega, USA) on the QuantStudio™ 7 Flex Real-Time PCR System (Applied Biosystems). For each reaction run, the specificity of the amplification was validated, and the threshold cycle above background was calculated using Bio-Rad iCycler; PCR efficiency was close to 100%.

The relative expression levels of the individual target genes were calculated using a modified double delta method [55]. Error bars of qRT-PCR data in all figures represent standard deviations, and the significance of differences was estimated at the *p* = 0.05 level using Student’s *t*-test.

### 4.10. Endogenous IAA Content Analysis

The main inflorescences and cauline leaves of 40-day-old transgenic and wild-type *Arabidopsis* plants were collected for IAA measurement. Three biological replicates were included, with five plants per replicate. IAA content was determined using liquid chromatography–mass spectrometry on the Agilent 160 Infinity-6420 (Agilent Technologies) platform by Tsingtao Sci-Tech Innovation Co., Ltd. (China).

### 4.11. Phenotypic and Statistical Analyses

To evaluate the effects of *BnERF114.A1*, the plant height, length of the main inflorescence, and number of primary branches in transgenic and wild-type plants were determined at 30, 37, 44, and 51 days after planting. The number of siliques per plant, number of seeds per silique, seed yield per plant, and biomass per plant were evaluated at maturity. The traits were assessed in 3–10 randomly selected transgenic or wild-type plants. Statistical analysis was performed using unpaired *t*-test or one-way ANOVA to evaluate significant differences.

## Supplementary Materials

The following supporting information can be downloaded at: https://www.mdpi.com/article/10.3390/ijms23042210/s1.

## Abbreviations

ERF	ETHYLENE RESPONSE FACTOR
SAM	shoot apical meristem
AM	axillary meristem
CK	cytokinin
IAA	indole-3-acetic acid
AP2	APETALA2
PIN	PIN-FORMED
ABCB	ATP-binding cassette B
PGP	P-glycoprotein
aa	amino acid
JA	jasmonic acid
MES	monosulphuron ester sodium
CTAB	cetyltrimethylammonium bromide
CDS	coding sequence
qRT-PCR	quantitative real-time polymerase chain reaction
