# Supplementary material for: BnERF114.A1, a Rapeseed Gene Encoding APETALA2/ETHYLENE RESPONSE FACTOR, Regulates Plant Architecture through Auxin Accumulation in the Apex in Arabidopsis
Source: Int J Mol Sci. 2022 Feb 17;23(4):2210. doi: 10.3390/ijms23042210 (PMC8877518; doi:10.3390/ijms23042210)
Supplement: Supplementary file 1 [file ijms-23-02210-s001.zip › Table S1.pdf]

Table S1. Primers used in this study.

Primer Name	Primer Sequence（5’-3’）	Purpose
BnERF114.A1-F	ATGGATCCTCGATACAATGGGAA	CDS cloning
BnERF114.A1-R	ACTAGTATAACCCGAATGTGGAGAGGAAG	
114pro-F	GGAATTCATTTAGCCGTTGCAGCATTT	Gus analysis
114pro-R	CGGATCCATTTTGCTAGATTTGATTTAAAGTCC	
114SL-F	CTGAATTCATGGATCCTCGATACAATGGGAA	Subcellular localization
114SL-R	CTGACTAGTATAACCCGAATGTGGAGAGGAAG	
E756-F	CATATGATGGATCCTCGATACAATGGGAA	Transcription activity
E756-R	GAATTCCTAATAACCCGAATGTGGAGAGGAAG	
E246-F	CATATGGATCCTCGATACAATGGG	Transcription activity
E246-R	GAATTCCTATCTCGTCCTCAAGTTCCCTTG	
E177-F	CATATGCATTATAGAGGGGTAAGGCAAAG	Transcription activity
E177-R	GAATTCCTATTCAGGGAAGTTGAGTTTGGC	
E333-F	CATATGAGGGTTCAGCTAGGAAGCAATAT	Transcription activity
E333-R	GAATTCCTAATAACCCGAATGTGGAGAGGA	
BnUBC21-qF	CCTCTGCAGCCTCCTCAAGT	Reference gene
BnUBC21-qR	CATATCTCCCCTGTCTTGAAATGC	
BnERF114.C2-qF	CCTTTGGAGGCGAGGAAAATGAA	Expression level
BnERF114.C2-qR	CGTGAGAGCTGCCTTGTTGGTTA	
BnERF114.A1-qF	AAGATGAGAACTTATTCCCTGTC	Expression level
BnERF114.A1-qR	TTGACTAGGGTCTTGTGGATT	
BnERF114.A6-qF	GAAATGGGCAGCTGAAATACGG	Expression level
BnERF114.A6-qR	GATAACTGATCATTATAGGCAACG	
BnERF114.C3-qF	CAAGATCAAGGAAACTCGATGG	Expression level
BnERF114.C3-qR	GGTAGAGTTTGATAGCTGATCATTAC	
AtUBC21-qF	CTGCGACTCAGGGAATCTTCTAA	Reference gene
AtUBC21-qR	TTGTGCCATTGAATTGAACCC	
AUX1-qF	GCACTTCTCGACCACTCC	Expression level
AUX1-qR	TCCCAATCACTTTCTCCC	
LAX1-qF	GCTGTGACCGTGGAAATA	Expression level
LAX1-qR	ACGGAAGCGTTAATGTGA	
LAX2-qF	ATTGTTGGATTCGGGTTCG	Expression level
LAX2-qR	GGTGGTGGGCATTGGTAG	

LAX3-qF	TGCTTACCTTTGCTCCTG	Expression level
LAX3-qR	AACCCAACTACGAATACCC	
PGP1-qF	TGGCTGCTCTTTACCTCTGT	Expression level
PGP1-qR	CTCCACATCCAACACGAAAT	
PGP2-qF	ATCGCACGCTAAGCAGAC	Expression level
PGP2-qR	ACGCCATACATCCAGTCC	
PGP4-qF	AGACAAGCAGCGAGAATA	Expression level
PGP4-qR	CTATCACAAAGCCTCCTAC	
PGP19-qF	CTTGTGAGTAAAGGCGTGTC	Expression level
PGP19-qR	GGGTCAATCCTGGTCTGC	
PIN1-qF	ACGGCTCTGTCAAATGGT	Expression level
PIN1-qR	TGTTAGCGGCGATGAAGT	
PIN2-qF	TCACTATCAACACTGCCTAA	Expression level
PIN2-qR	CACGGAACTCAAACAAGA	
PIN3-qF	GGCTGCTTCCATTGTTTC	Expression level
PIN3-qR	TTCCCGTCGTCACCTATC	
PIN4-qF	CTTGTGGGAACTCTGTCG	Expression level
PIN4-qR	AGTGCTTAGAATCGTGGG	
PIN5-qF	TATGGCTCTGTGAAATGG	Expression level
PIN5-qR	AAGACCGTGACTATGATGAC	
PIN6-qF	TCCGTCAACACTCCTAATAAC	Expression level
PIN6-qR	ATCCGAACCCGCCTAAAT	
PIN7-qF	CACAGCAGAGCTAAACCCTA	Expression level
PIN7-qR	TCCCACCTGAAAGCAACA	
PIN8-qF	ATCCAAATACATACGCAACA	Expression level
PIN8-qR	ATTCCTAAGCCTCCATCA	
YUCCA1-qF	CGGTCGGATTCAATAGCATCTC	Expression level
YUCCA1-qR	AAGCGTAGGACTCAAGGTAGG	
YUCCA2-qF	GGATGAGACAATGGAGTATG	Expression level
YUCCA2-qR	ATATTTCACCGCTCTTATAGG	
YUCCA4-qF	ACGCATCTGGTCTATGGAATG	Expression level
YUCCA4-qR	CGGACTTGTACGCACTGG	
YUCCA6-qF	GGTTGAGTCGGCTGCGTTTG	Expression level
YUCCA6-qR	ACATACTCCGTCGTGCCTTCTTC	
