# Supplementary material for: BnERF114.A1, a Rapeseed Gene Encoding APETALA2/ETHYLENE RESPONSE FACTOR, Regulates Plant Architecture through Auxin Accumulation in the Apex in Arabidopsis
Source: Int J Mol Sci. 2022 Feb 17;23(4):2210. doi: 10.3390/ijms23042210 (PMC8877518; doi:10.3390/ijms23042210)
Supplement: Supplementary file 1 [file ijms-23-02210-s001.zip › Table S2.pdf]

**Table S2. Amino acid alignment of AtERF114 and its orthologues ERF114s in *B. napus*, *B. rapa*, and *B. oleracea*.**

| Protein Name | Protein ID | Identity with AtERF114 | Query Coverage | E-Value |
|---|---|---|---|---|
| BnERF114.A1 | XP_013727956.1 | 76% | 99% | 2e-114 |
| BnERF114.C2 | XP_013675388.1 | 77% | 99% | 2e-110 |
| BnERF114.A6 | XP_013645692.1 | 74% | 97% | 9e-91 |
| BnERF114.C3 | XP_013718191.1 | 71% | 97% | 5e-91 |
| BrERF114.A2 | XP_009130180.1 | 77% | 99% | 2e-110 |
| BrERF114.A6 | XP_018515123.1 | 73% | 99% | 4e-91 |
| BoERF114.C2 | XP_013626888.1 | 71% | 97% | 2e-89 |
| BoERF114.C6 | XP_013619319.1 | 79% | 99% | 5e-113 |

NB: BnERF114.A1, BnERF114.C2, BnERF114.A6, and BnERF114.C3 are ERF114 proteins in *B. napus*; BrERF114.A2 and BrERF114.A6 are ERF114 proteins in *B. rapa*; BoERF114.C2 and BoERF114.C6 are ERF114 proteins in *B. oleracea*.
