# Supplementary figures and images for: BnERF114.A1, a Rapeseed Gene Encoding APETALA2/ETHYLENE RESPONSE FACTOR, Regulates Plant Architecture through Auxin Accumulation in the Apex in Arabidopsis
Source: Int J Mol Sci. 2022 Feb 17;23(4):2210. doi: 10.3390/ijms23042210 (PMC8877518; doi:10.3390/ijms23042210)

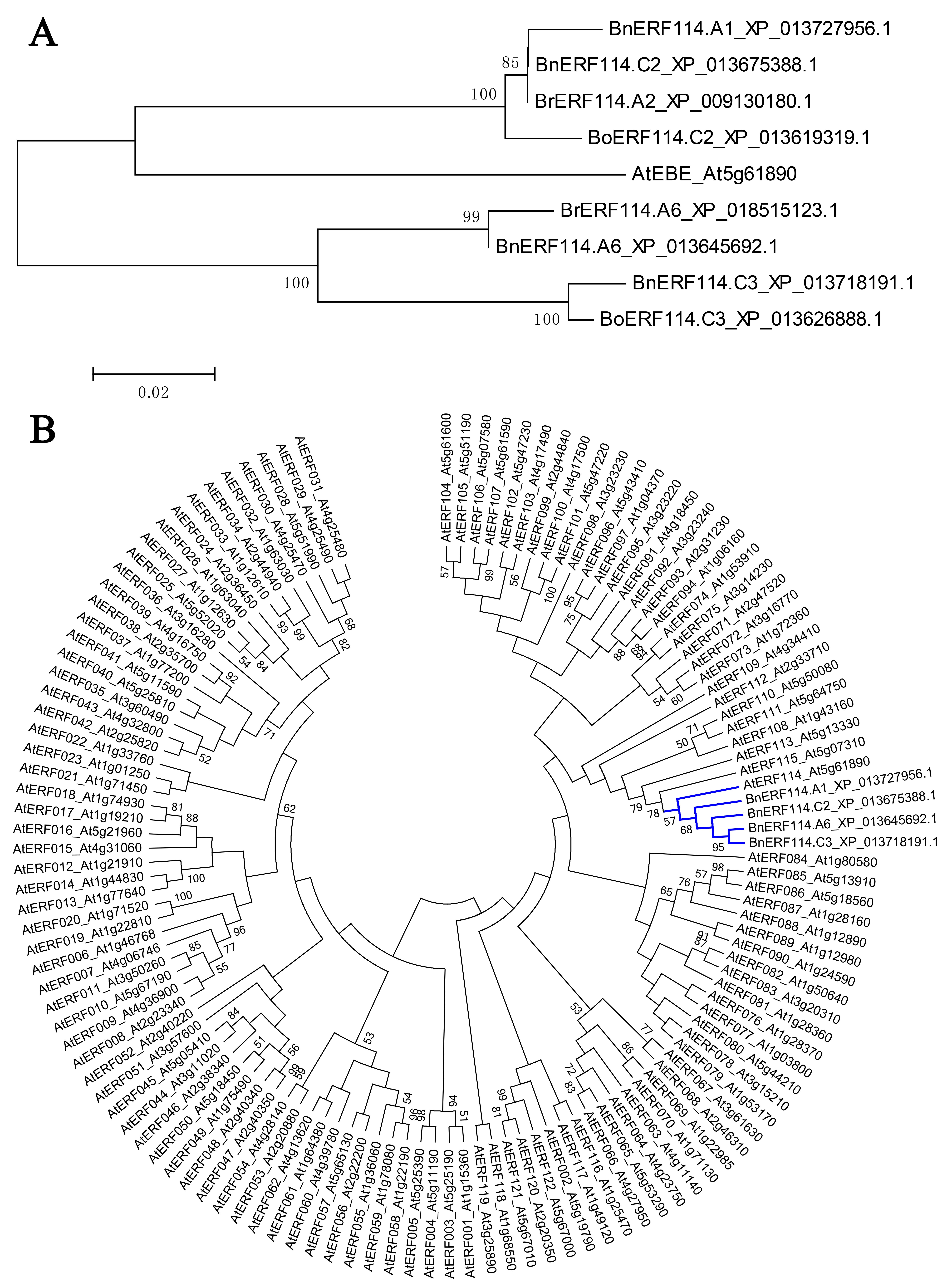

Supplement: Supplementary file 1 [file ijms-23-02210-s001.zip › Figure S1.jpg]

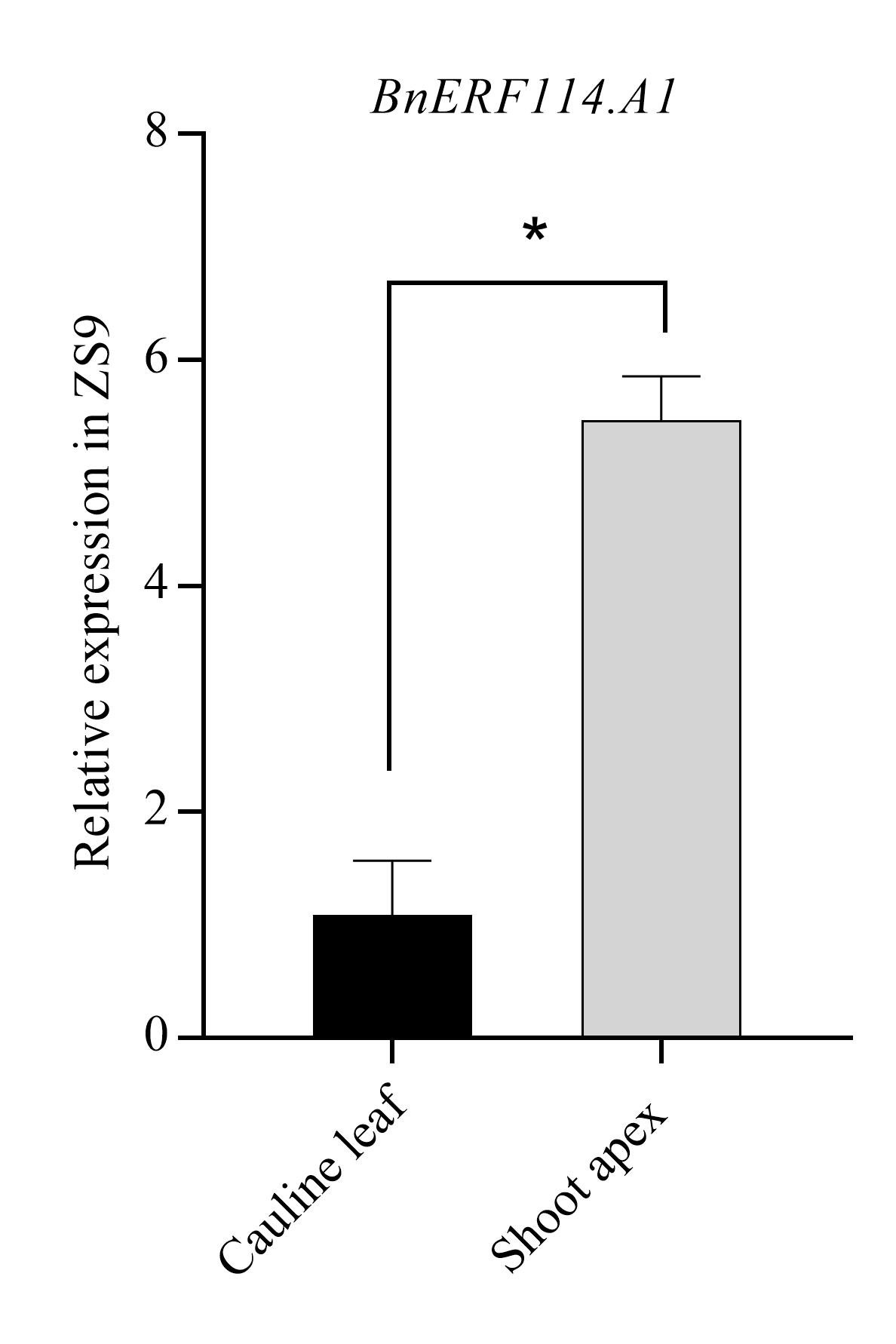

Supplement: Supplementary file 1 [file ijms-23-02210-s001.zip › Figure S2.jpg]

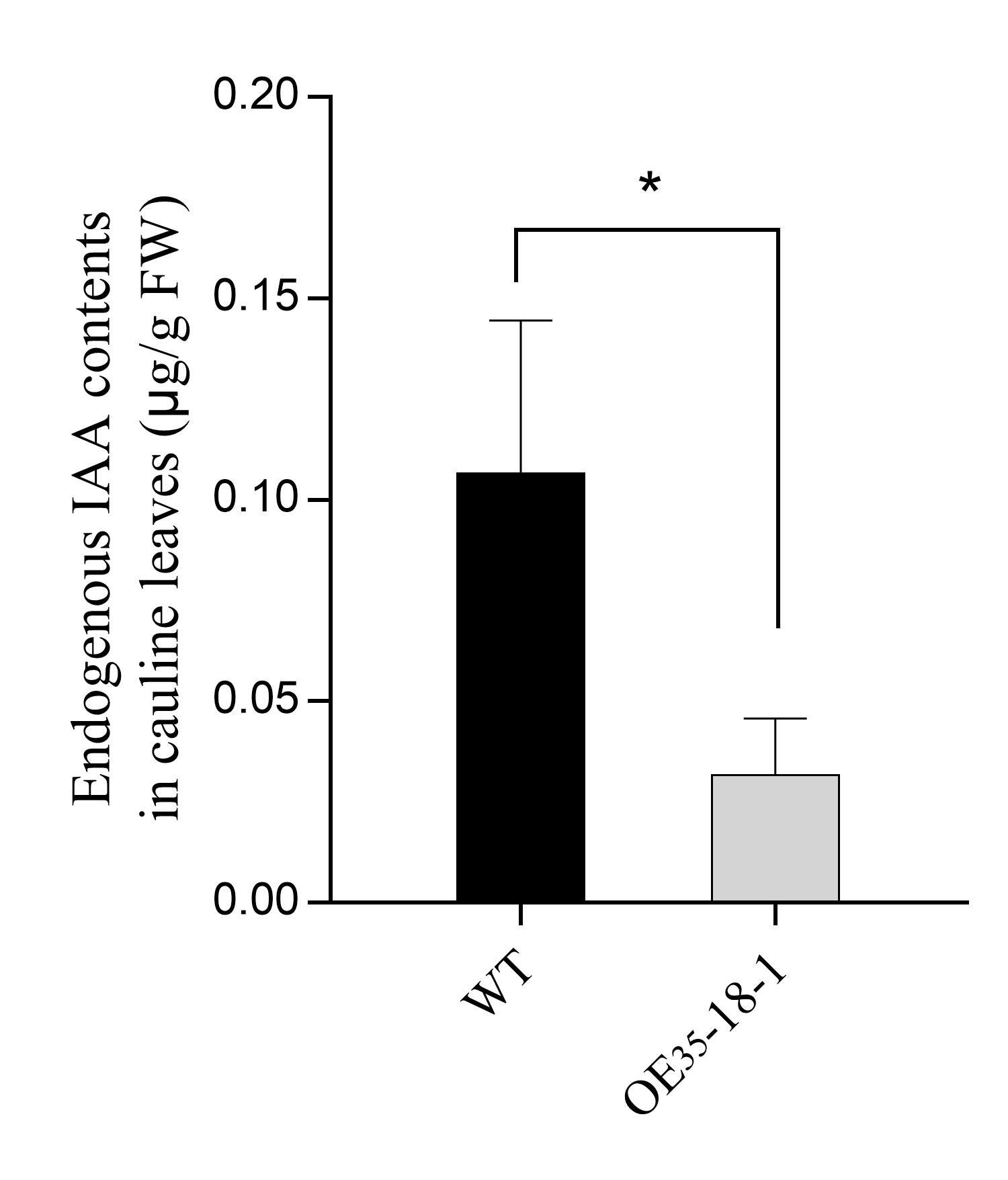

Supplement: Supplementary file 1 [file ijms-23-02210-s001.zip › Figure S3.jpg]
